# Normalizing flow based neural processes for Alzheimer’s disease progression prediction

**DOI:** 10.1371/journal.pone.0345958

**Published:** 2026-04-20

**Authors:** Emad Al-anbari, Hossein Karshenas, Bijan Shoushtarian

**Affiliations:** Artificial Intelligence Department, Faculty of Computer Engineering, University of Isfahan, Isfahan, Iran; Instituto Tecnológico y de Estudios Superiores de Monterrey: Tecnologico de Monterrey, MEXICO

## Abstract

As one of the most common neurodegenerative diseases, Alzheimer’s accounts for serious health problems worldwide. Accurate detection and prediction of this disease contribute to the health system for better prevention and interventions in the treatment plans. However, traditional models designed for prediction and classification face several challenges, including handling complex data, which neglects many data points for the diagnosis. To overcome this challenge, we propose a novel model based on the integration of Neural Processes (NPs) and Normalizing Flows (NFs). The dataset used for this study is the Alzheimer’s Disease Prediction of Longitudinal Evolution (TADPOLE). We selected various features to build an efficient model, including cognitive, neuroimaging, genetic, and demographic data. which contains three classes: Cognitively Normal (CN), Mild Cognitive Impairment (MCI), and AD. The proposed model is able to capture the temporal dependencies present in the complex distribution. The stochastic processes were modeled by NPs, while NF was able to transform the Gaussian distributions from simple to complex distributions, allowing them to model a wide range of data distributions. The prediction performance and robustness have been enhanced since this framework is able the adapt to every patient trajectory and generalizing across different populations. The model was compared with other models, such as SNP, deep geometric learning, Manifold DCNN, and other models. Our model (SNP-NF) made an improvement regarding mAUC, Precision, and Recall, approximately 3%,1%, and 0.7%, respectively from our previous model, which utilized only NP. These results demonstrate the capability of the proposed approach to provide early detection and personal treatment plans for patients suffering from this disease.

## Introduction

Alzheimer’s Disease (AD) is a progressive neurodegenerative disorder that profoundly impacts cognitive functions, memory, and behavior. Accurate prediction of AD progression can transform clinical management by enabling early intervention and personalized care strategies. Early prediction facilitates not only pharmaceutical interventions but also psychosocial adjustments for patients and caregivers. Despite the critical need, the inherent complexity of AD progression, driven by multifactorial and individual-specific paths, makes prediction highly challenging [1,2].

Modeling AD progression remains inherently challenging due to its highly individualized, non-linear trajectories influenced by a combination of genetic, clinical, and environmental factors. Traditional machine learning [3] and deep learning methods [4,5] including convolutional neural networks (CNNs) [6], recurrent neural networks (RNNs) [7], and ensemble models [8] have demonstrated promise in analyzing AD-related data. Yet, these approaches often face limitations such as overfitting, low interpretability, and dependence on large, well-annotated datasets. Furthermore, the performance of classic models is greatly affected by the original distribution of the data. With the AD, the data characteristics are typically complex and of high dimension, even far from a Gaussian distribution, leading to the difficulty in accurate prediction. Furthermore, since AD data is generally distributed in its native feature scale, which is often not assumed to be Gaussian, classifying a reliable pattern could cause misclassification, i.e., these abstracted models cannot learn the distinctive subtle non-linear property of these data types.

Neural Processes (NPs) [9] represent a family of models that combine the strengths of deep neural networks with the flexibility of probabilistic modeling. They are designed to learn distributions over functions from observed data. They are particularly effective in few-shot learning, meta-learning, and modeling uncertainty in tasks such as regression and contextual bandits. Central to their operation is the notion of learning a latent representation of data, from which predictions about unseen inputs can be made in a flexible, data-driven manner. However, traditional NPs, such as Conditional Neural Processes (CNPs) and their stochastic variants like Neural Process (NP), often rely on relatively simplistic assumptions about the structure of the latent space. Typically, these models assume that the latent variables are drawn from a multivariate Gaussian distribution with fixed or learned parameters. While computationally efficient, this assumption may limit the expressiveness of the latent space and hinder the model’s ability to capture complex posterior distributions, especially in tasks that involve multimodal or highly structured uncertainty.

We introduce a new framework, which combines NPs, a probabilistic meta-learning method, with NFs, to improve the representation and distributional modeling power of predictive models. NPs are used for their ability to learn uncertainty-aware, context-dependent representations from a small set of data, which enables the model to generalize well across various tasks with few supervision signals. However, classical NPs typically use simple distributional assumptions, such as Gaussianity, which may not be able to describe the real complexity of the actual data, particularly in biomedical tasks such as Alzheimer’s Disease prediction. Traditional ML models often require large labeled datasets and lack reliable uncertainty estimation, limiting their adaptability to unseen data. NPs address these issues by combining neural networks with probabilistic inference, allowing them to learn functions from a few examples while quantifying prediction uncertainty. Meanwhile, Normalizing Flows (NFs) overcome the expressivity limitations of conventional probabilistic models by enabling exact likelihood estimation and flexible transformation of complex, multimodal data distributions.

Traditional machine learning models often rely on fixed parametric assumptions and lack the ability to effectively quantify uncertainty, adapt to new tasks with limited data, or model highly complex and multimodal data distributions. These limitations restrict their performance in domains requiring reliable probabilistic reasoning and flexible representation learning. NPs address these challenges by combining the strengths of neural networks and Gaussian processes, enabling efficient uncertainty estimation and few-shot learning through conditional distribution modeling. Similarly, Normalizing Flows (NFs) provide a powerful generative framework capable of exact likelihood estimation and invertible mapping between complex data distributions, allowing them to model non-Gaussian or high-dimensional patterns that traditional ML models fail to capture. Together, NPs and NFs offer a data-efficient, probabilistic approach that directly overcomes the rigidity, poor calibration, and limited adaptability of conventional ML architectures.

To address this limitation, normalizing flows have emerged as a powerful tool for constructing flexible and tractable distributions. Normalizing flows transform simple base distributions (e.g., Gaussian) into more complex ones through a sequence of invertible mappings. This framework enables the modeling of highly expressive latent distributions while retaining the ability to compute exact likelihoods and perform efficient sampling. Incorporating normalizing flows into the latent space of Neural Processes offers the potential to overcome the rigidity of Gaussian priors and better capture the diversity and structure of real-world data. This combination enables richer posterior approximations, improved uncertainty quantification, and more accurate predictive performance in downstream tasks.

The implications of the combination of the model realizes high accuracy predictions by more closely reflecting underlying data distribution functions; the uncertainty estimations are reliable, which can be beneficial to clinical decisions. It is scalable and data-efficient, especially in scenarios where labeled data is scarce. The hybrid framework’s potency is shown empirically with better performance, with robust uncertainty quantification, and a stronger generalization to heterogeneous data, firmly establishing a new state-of-the-art in predictive modeling for complex, high-stakes domains.

This paper makes the following contributions:

1. Introduces a hybrid framework leveraging Neural Processes and Normalizing Flow for AD progression prediction.
2. Provides a detailed architectural description of the proposed model, including its probabilistic and sequential learning components.
3. Validates the model on real-world datasets and benchmarks it against state-of-the-art methods.
4. Explores the interpretability and robustness of the model in predicting diverse AD progression trajectories.

The rest of this paper is organized as follows: Related work’s section‌‌ reviews the field of different pipeline approaches that handle AD prediction. the proposed model is presented next. The simulation results and comparison with state-of-the-art techniques are discussed in the results section. Finally, a conclusion of the proposed method and findings are outlined in the last section.

## Related work

### Statistical methods

Classical statistical techniques have been instrumental in understanding and predicting the progression of AD. Epidemiological studies have employed logistic regression to identify significant risk factors, such as age and the APOE-ϵ4 allele, while Cox proportional hazards models have provided insights into time-to-event data, particularly the progression from mild cognitive impairment (MCI) to Alzheimer’s disease [10]. Longitudinal data analysis using linear mixed-effects models has allowed researchers to track cognitive decline through measures like the MMSE and CDR scales, highlighting the progression variability among individuals [11].

Survival analysis methods, such as Kaplan-Meier estimators and competing risk models, have further enhanced the understanding of progression patterns, distinguishing between dementia onset and other events like mortality [12].

Cross-sectional studies using ANOVA and PCA have revealed critical insights into structural and functional brain changes, offering a better grasp of disease stages through biomarker and neuroimaging data [13]. Diagnostic frameworks leveraging discriminant analysis and logistic regression have integrated cognitive and biomarker data to improve diagnostic accuracy, particularly with the inclusion of cerebrospinal fluid biomarkers such as amyloid-β and tau [14]. In biomarker analysis, regression techniques have explored the relationships between biomarkers and cognitive outcomes, while ROC curve analysis has evaluated their diagnostic utility [15]. Neuroimaging studies, employing voxel-based morphometry, ROI analyses, and statistical parametric mapping, have identified brain regions significantly affected by Alzheimer’s progression, linking these changes to clinical measures [16]. Despite their contributions, classical statistical methods face challenges with high-dimensional datasets and assumptions of linearity, which are often violated in complex biological systems. The combination of these conventional methods with advanced machine learning technology is expected to eliminate these limitations and yield new insights into the progressive evolution of Alzheimer’s disease. Thus, classical models can complement modern computational paradigms and improve the predictive models developed for a more clarifying understanding of disease, enhancing its clinical end results and treatment schemes.

### Gaussian processes approaches

Due to the accuracy of capturing complex temporal and spatial correlations, Gaussian Processes (GPs) have demonstrated a high impact on the field of prediction as a statistical tool. Furthermore, since they perform as non-parametric Bayesian, GPs excel in uncertainty quantification, making them particularly suited to addressing the variability inherent in AD. These models were also widely proposed in analyzing clinical and biomarker data, including cerebrospinal fluid amyloid and tau levels, alongside cognitive assessments such as the Mini-Mental State Examination [17]. Moreover, GPs have been employed for personalized prediction models since these models can follow clinicians to estimate transitions from mild cognitive impairment to dementia. Besides that, an advanced version of GP namely a multi-task GP model, has further enhanced the prediction accuracy by jointly analyzing multiple biomarkers, offering a high potential in improving AD prognosis and treatment planning.

Apart from the clinical and biomarker data, successful applications of GPs are also concerned with neuroimaging, including MRI and PET scans, to model structural and functional changes in the brain related to AD progression. This technique might serve well in high-dimensional neuroimaging data, especially those pretreatments that were modeling the localized prediction of the disease progress through the linkage of brain atrophy related to cognitive decline [18]. Furthermore, GPs are adept at integrating multi-modal data sources, such as genetic, clinical, and imaging data, which improve predictive accuracy and robustness. For example, multi-modal GP models have been used to combine genetic risk scores, such as APOE allele status, with imaging biomarkers to predict AD onset [19]. Computational challenges with large datasets advance in sparse approximations, and deep GP frameworks hold promise for future applications once integrated with machine learning to improve scalability and predictive capabilities [15]. While classical statistical techniques have provided valuable insights into Alzheimer’s disease progression, they have certain limitations. Many methods assume linear relationships and normality, which may not always hold in complex biological data. Additionally, classical models often struggle with high-dimensional datasets, such as those derived from genomics or advanced neuroimaging, necessitating dimensionality reduction or variable selection techniques.

### Machine and deep learning models

Machine learning (ML) approaches have significantly advanced the understanding and prediction of AD progression. Supervised learning methods, including support vector machines (SVMs), random forests (RFs), and ensemble techniques, have demonstrated efficacy in classifying stages of AD and predicting disease trajectories. Models trained on neuroimaging and biomarker data, such as those incorporating MRI and cerebrospinal fluid (CSF) features have achieved high diagnostic accuracy [20]. Neural network-based methods, particularly convolutional neural networks (CNNs) have excelled in extracting spatial patterns from imaging data, while recurrent neural networks (RNNs) and long short-term memory (LSTM) networks have effectively modeled temporal progressions in clinical scores and biomarkers [19,21]. Unsupervised learning techniques, such as principal component analysis (PCA), t-distributed stochastic neighbor embedding (t-SNE) and clustering algorithms have identified novel subtypes and phenotypes within AD cohorts, facilitating stratified analyses and targeted interventions [15,22]. Furthermore, the integration of multimodal data through deep learning models has achieved state-of-the-art results, combining genetic, imaging, and clinical information into comprehensive predictive frameworks [23].

In addition to strengthening diagnostic capabilities, ML models have tackled significant problems such as explainability and translation into clinical practice. Tools such as Shapely Additive explanations (SHAP) and Local Interpretable Model-Agnostic Explanations (LIME) have also substantially lent interpretability to movement in the understanding of ML prediction results, enabling the clinician to adopt these models in the decision-making process [24]. Additional challenges have been raised in the field of prediction, including data heterogeneity, missingness, and model generalizability still exist. Federated and transfer learning techniques have shown promising results, enabling collaborative training across various datasets while maintaining privacy [25]. Moreover, novel frameworks such as transformers have been adapted for sequential data analysis, surpassing traditional RNNs in capturing complex dependencies and interactions [26].

### Neural processes

Neural Processes represent a class of models that blend the strengths of neural networks and stochastic processes by learning distributions over functions. Instead of training a neural network from scratch for each new task, a Neural Process observes a small “context” set of input‐output pairs and infers a predictive distribution for any new input conditioned on that context. This means the model can quickly adapt to new observations and estimate uncertainty in its predictions like a Gaussian Process but with the computational efficiency and flexibility of neural networks [9]. Recent research has emphasized combining NPs with deep learning models to enhance classification and prediction performance and model interpretability. For example, Kim et al. [27] incorporated latent variables, enabling NPs to conduct global function sampling by adjusting their priors based on contextual data during testing. However, it is also demonstrated that these processes remain susceptible to underfitting the observed context set to which they have been conditioned. Nguyen et al. [28] propose substituting the latent variables with fully attentive Transformer designs. The resulting Transformer Neural Processes (TNPs) approach provide uncertainty-aware meta-learning as a sequence modeling task, integrating Transformers into the NP framework through three primary processes. The sequence modeling approach entails treating all input points as an ordered sequence, wherein the predictive likelihood of the target points is modeled autoregressively.

Emad Alanbari et al. [29] presented a framework that combines NPs and a transformer encoder to simulate the intricate temporal relationships seen in longitudinal health data. The model can identify patterns and variances that point to the advancement of a disease. The method is innovative because it combines transformer designs, which are recognized for their capacity to capture long-range relationships, with NPs, which are well-known for their capacity to describe stochastic processes. This combination improves the predictive ability and resilience of the model by allowing it to generalize across a variety of populations and successfully adjust to the trajectories of individual patients.

### Normalizing flows

NFs have become groundbreaking models in probabilistic modeling, providing highly expressive frameworks for classification challenges. By deploying a sequence of invertible transformations to simple base distributions, NFs enable precise density estimation alongside exact likelihood estimations. Recent research has emphasized combining NFs with deep learning models to enhance classification performance and model interpretability. For example, Smith et al. [30] proposed conditional normalizing flows, which have shown significant potential in improving classification performance on high-dimensional datasets by capturing complex dependencies. Moreover, researchers have leveraged NFs for uncertainty quantification, as a crucial capability for safety-critical domains, including medical diagnosis and autonomous systems [31].

NFs can also be merged with other probabilistic models to enhance scalability and adaptability. Zhang et al. [32] present integrating the NFs model with Gaussian processes to improve classification performance in sparse data scenarios. Furthermore, the NFs framework has been tailored to address multi-class classification challenges effectively, employing techniques including augmented normalizing flows, as presented in [33], where augmented normalizing flows have demonstrated potential in handling imbalanced datasets and refining decision boundaries. These advancements highlight the flexibility of NFs in tackling contemporary classification challenges, opening avenues for applications in fields such as finance, healthcare, and robotics.

To estimate the optimum value of prediction accuracy for the AD classification, we propose an incorporation of NFs in the NP framework, namely as an SNP-NF model. By incorporating NF with the model, the encoder can map the input to a latent space, where complex dependencies can be modeled, which the decoder can then use to reconstruct or generate data more effectively. NF can refine the posterior approximation by making it more expressive than the typical Gaussian assumption. It will lead to better optimization and more accurate latent representations of the final results.

## The method

### Problem statement

This study aims to develop an advanced predictive model for Alzheimer’s disease (AD) progression by leveraging longitudinal patient data, including brain imaging, cognitive function assessments, and demographic features. Given a time series of observations for each patient, the objective is to predict the patient’s future diagnostic stage.

- Let $x_{i,j}\in {\mathbb{R}}^{d}$ denote the *d*-dimensional feature vector from the *i*-th visit of the *j*-th patient, encompassing cognitive, imaging, and demographic data.
- Let $y_{i,j}\in \{0,1,2\}$ represent the diagnostic label for the visit, where:
  – *y* = 0: Cognitively Normal (CN),
  – *y* = 1: Mild Cognitive Impairment (MCI),
  – *y* = 2: Probable Alzheimer’s Disease (AD).

For modeling, we reshape the input data into sequences with a sliding window of size *k*:


$$x_{i,j}^{\prime }=\left[(x_{i,j},y_{i,j}),\ldots ,(x_{i+k,j},y_{i+k,j})\right],\;y_{i,j}^{\prime }=y_{i+k+1,j}.$$ (1)

Here, $x_{i,j}^{\prime }$ is the context sequence (historical visits), and $y_{i,j}^{\prime }$ is the target label (future diagnosis).

The goal is to learn a predictive function $f_{\theta }$, parameterized by θ, that maps each input sequence to its corresponding future diagnostic label:


$$f_{\theta }(x_{i,j}^{\prime })\approx y_{i,j}^{\prime }=y_{i+k+1,j}.$$ (2)

The model must capture temporal dependencies in the patient’s trajectory, integrating Transformer-based encoding with neural processes (NPs) and normalizing flows (NFs) to robustly model context-target relationships for accurate prediction.

### The proposed sequential neural process with normalizing flow

Rather than focusing on the labeling classification strategy only, or the spatial patterns from longitudinal data as mentioned in previous works, the proposed attention mechanism in the NPS-NF model will prioritize critical features, enabling robust pattern recognition in noisy datasets. On the other hand, NF augments the model by transforming simple distributions into complex ones, allowing for precise density estimation among the resulting features of the decoder. This advantage is particularly valuable for AD, where progression trajectories exhibit significant inter-patient variability. Furthermore, the Transformer encoder is then leveraged to capture long-range temporal dependencies in the digital data. Moreover, the proposed NPs are designed to learn stochastic processes, making them ideal for modeling the spatiotemporal variability of the AD datasets. By framing the prediction problem as a normal process, NPs provide predictive distributions, accounting for uncertainties in progression trajectories.

The main difference between Sequential Neural Process(SNPs) and Sequential Neural Process with Normalizing Flow(SNP-NFs) for AD prediction is their capacity to model uncertainty and data complexity. SNPs are a more flexible form of meta-learning for uncertainty across patients compared to the variants in this work. which however require simpler latent variable distributions. On the other hand, SNP-NF improves this by employing Normalizing Flows to model complicated non-Gaussian latent distributions. This results in a better coverage of the underlying data variability and of the data distribution, which in turn enhances the model’s predictive and generalization ability across diverse clinical patterns of Alzheimer’s progression.

#### Model architecture.

**Fig 1:**
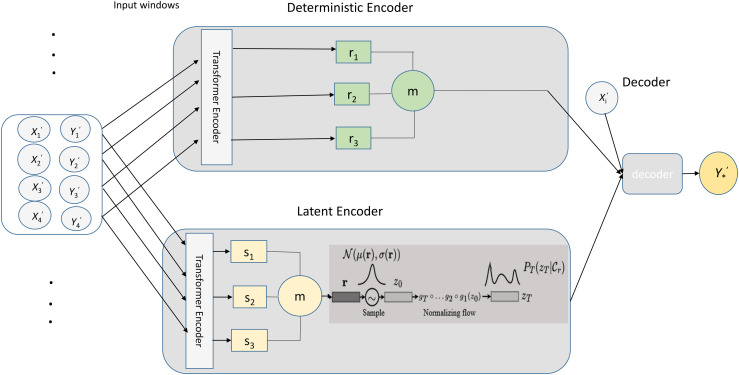
The proposed sequential neural process with Normalizing Flows structure.

The proposed SNP-NF model can achieve a more expressive latent variable representation, capturing complex data distributions. The framework comprises five main components, each contributing uniquely to the model’s overall learning capacity. Fig 1 illustrates the structure of the proposed model.

#### a. Input layer.

The model receives a set of input–output pairs ($x_{i},y_{i}$)organized as contextual windows. These serve as conditioning information for both encoder paths.

#### b. Deterministic Encoder.

The deterministic encoder employs a Transformer-based feature extractor to encode contextual relationships among inputs. Each encoded vector *r*_*i*_ represents localized patterns; these are aggregated by the mean operator m to form a deterministic representation *r*_*d*_, which captures shared structural information across contexts.

#### c. Latent Encoder.

The latent encoder uses a similar Transformer backbone but outputs a stochastic embedding *s*_*i*_. The aggregated latent representation s parameterize a latent distribution. A Normalizing Flow transforms a simple Gaussian prior into a complex posterior, enabling flexible modeling of multimodal uncertainties. This design allows the model to represent multiple plausible functional mappings rather than a single deterministic one.

#### d. The Decoder.

The decoder component integrates transformed latent variables with the contextual representations generated by the encoders. The decoder produces target predictions informed by contextual and transformed latent features by passing these representations through fully connected layers. This integration step is crucial, as it enables the model to leverage the enhanced expressiveness of the latent space in a way that directly improves target inference.

#### e. Output layer.

The final output y^*^corresponds to the model’s prediction or reconstruction for the target window, capturing both learned structure and uncertainty.

## Model training with normalizing flows

The loss function presented for the classifier model and regularization term is Kullback-Leibler (KL) divergence. Cross entropy loss:


$$S(y_{-t}\prime )=-\sum_{i}(y-t\prime )\log p(y_{predicted}\prime )$$ (3)

(KL) divergence:


$$\begin{array}{c} D_{KL}(y\prime_{-t}||(x_{context}\prime ,y\prime_{context}))=\\ \sum_{i}p_{A}(y\prime_{-t}(\log p_{A}(y\prime_{-t}-p_{A}(y\prime_{context})\log pB(𝐲\prime_{context}) \end{array}$$ (4)

$$Loss=S(y_{i}\prime )+D_{KL}(y_{i}\prime ||(x_{J}\prime ,y_{J}\prime ))$$ (5)

The cross-entropy is utilized to compute the classification loss, which compares the result of model predictions with the ground-truth labels. It confirms that the model successfully learned how to classify input instances. Moreover, the regularization KL divergence [34] supports the model distribution, denoted by *q*_*target*_ and *q*_*context*_, precisely indicating the target distribution. By penalizing deviations from the target distribution, the regularization enhances the output distribution, assisting the model’s generalization and alleviating the overfitting.

During training, the objective is to maximize the likelihood of observed data by adjusting the parameters of both the neural process and the normalizing flows. The flows are trained to transform the base distribution *p*(*z* − 0) into a distribution *p*(*z* − *K*) that better matches the underlying structure of the latent variables. It is achieved by minimizing the Kullback-Leibler divergence between the transformed and the target distribution, ensuring that the model learns an expressive latent representation that aligns well with the observed data. By applying these Planar Flow transformations within the neural process framework, the model gains the flexibility to adapt its latent space, capturing complex dependencies and non-Gaussian characteristics inherent in the data.

Our findings indicate that performance improves as the flow transformation depth increases, as evidenced by enhanced metrics across multiple experiments. This progression suggests the model better represents challenging data structures, such as one-to-many relationships, where a single context point could correspond to several plausible target outputs. Each transformation step refines the base distribution, enabling more nuanced modeling of these relationships and demonstrating the value of deeper flows for capturing intricate data patterns. Empirically, we observed that the setting provides a trade-off, delivering sufficient transformation depth to capture complex distributions without incurring prohibitive computational costs or overfitting. Furthermore, while not directly tested, the improved adaptability of the latent space suggests potential benefits for few-shot learning tasks. In scenarios with limited data, a more expressive latent representation could allow the model to better capture task-specific characteristics, improving its capacity to generalize with minimal examples. The results of the proposed model provide evidence that the enhanced flexibility enabled by normalizing flows may support robust performance in few-shot settings, where capturing complex, data-scarce distributions is key to accurate predictions.

The proposed NF is represented mathematically by Kullback-Leibler (KL) divergence, which provides a measure of divergence between two probability distributions, including *q*_1(*z*)_ and *q*_2(*z*)_, as follows:


$$KL(q1||q2)=Eq1[log(\frac{q1(z)}{q2(z)})]$$ (6)

Equation 6 is fundamental for training the model since it guides the alignment of the transformed latent space with the target data distribution. Furthermore, the distributions that undergo transformations via normalizing flows, including $z_{1}=f_{1}(z_{0})$ and $z_{2}=f_{2}(z_{0})$, the transformed probability densities are represented by:


$$q_{1}(z_{1})=\frac{p_{1}(z_{0})}{\left|det(\frac{dz_{1}}{dz_{0}})\right|}$$ (7)

$$q_{2}(z_{2})=\frac{p_{2}(z_{0})}{\left|det(\frac{dz_{2}}{dz_{0}})\right|}$$ (8)

Thus, the KL divergence for flow-transformed distributions is expressed as:


$$KL(q1||q2)=E_{p1}[log\frac{p_{1}(z_{0})}{p_{2}(z_{0})}+log\frac{\left|det(\frac{dz_{2}}{dz_{0}})\right|}{\left|det(\frac{dz_{1}}{dz_{0}})\right|}]$$ (9)

Equation 9 ensures that the flow transformations are optimal in the learning process, allowing the model to learn mappings that align the transformed distributions with the target latent space.

## The results

In the following subsections, we will present the experiential settings, data preparations, evaluation metrics, experimental results, and discussion of the proposed model.

### Experimental settings

We used the Adam optimizer [35] to train the model parameters and set the initial learning rate to 10^−5^. Adam is popular in the deep learning community because of its learning rate updates and momentum selection that allow for expedient traversal of complex, high-dimensional optimization landscapes. Its capability of adapting learning rates to parameters allows for stabilizing training and speeding up convergence. We found the learning rate, as used in our experiments, by playing around in an iterative manner where we looked at trying to balance the rapid convergence of the model against the stability of the training process, the ideas being that we still want generally consistent and effective parameter updates to occur from epoch to epoch.

The dataset was divided into 80%–20% ratio for training and testing for model evaluation. This practice is common in machine learning and statistical modeling, providing a tradeoff between maximizing the data for the training process and maintaining a large enough test set to evaluate generalization. We tuned the rest of the hyperparameters, including the number of layers and embedding dimension. These were chosen based on a mix of computational practicality and empirical tuning to maximize model quality. A detailed list of hyperparameters is provided in Table 1.

**Table 1:** Hyperparameters.

Parameter	Value
Epochs	10
Time series length	4
Embedding dim	128
Number of encoding layers	2
Learning rate	10^−5^
Train test split	80% − 20%

### Data preparations

In the proposed model, we utilized the TADPOLE dataset [36]. This dataset, established in 2003 as a public-private partnership led by Dr. Michael W. Weiner, aims to determine whether serial imaging techniques such as magnetic resonance imaging (MRI) and positron emission tomography (PET), combined with biological markers and clinical and neuropsychological assessments, can predict the progression of mild cognitive impairment (MCI) and early Alzheimer’s disease. The TADPOLE dataset is aggregated data from three Alzheimer’s Disease Neuroimaging Initiative (ADNI) phases: ADNI 1, ADNI GO, and ADNI 2, providing a robust dataset for studying Alzheimer’s progression.

The TADPOLE dataset consists of data from 1,737 participants (975 males and 780 females) collected over 12,741 visits at 22 time points between 2003 and 2017 [37]. It includes 1,500 biomarkers, offering a comprehensive resource for developing predictive models. We selected various features to build an efficient model, including cognitive, neuroimaging, genetic, and demographic data. These extracted features can capture different aspects of the disease and are critical for understanding its progression. From the cognitive domain, we included widely used clinical metrics such as the Clinician Dementia Rating Scale Sum of Boxes (CDRSB), Alzheimer’s Disease Assessment Scale (ADAS11 and ADAS13), and Mini-Mental State Examination (MMSE). Furthermore, we incorporated neuropsychological test results from the Rey Auditory Verbal Learning Test (RAVLT), which evaluates verbal memory and learning abilities. These features are critical for tracking cognitive decline and the severity of dementia. The derived neuroimaging characteristics of MRI scans were also integral to our proposed model. These characteristics included volumes of the ventricles, hippocampus, whole brain, entorhinal cortex, and the midtemporal lobe. The above measurements provide critical insights into neurodegeneration and the structural brain changes associated with Alzheimer’s disease. Demographic features such as Roster ID and EXAMDATE were also included to organize the data further and provide temporal context. The diagnostic classification (DX) indicates that each participant’s diagnostic status can be used as the target variable for prediction.

To capture the temporal progression of Alzheimer’s disease, we transformed the dataset into time series representations for 13 selected features, including ID, EXAMDATE, CDRSB, ADAS11, ADAS13, MMSE, RAVLT, FAQ, ventricles, hippocampus, whole brain, entorhinal cortex, and the midtemporal lobe. A window length of four visits was used for each series separately. Thus allowing the model to analyze temporal patterns effectively. To ensure accurate training for the model, the participants with fewer than four visits were excluded from the experiments. This step is included to ensure adequate longitudinal data for analysis. The preprocessing steps ensured the independence of the training and testing data, thus improving the robustness and generalizability of the model. As mentioned, the dataset was divided into 80% for training and 20% for testing sets to evaluate model performance. Since the features have been carefully selected and transformed, we established a dataset that effectively captures the multidimensional and temporal aspects of Alzheimer’s disease progression, and sets the stage for building a predictive model.

### Evaluation metrics

The proposed model was evaluated with three metrics, including mean Area Under the Curve (mAUC), Precision, and Recall. These metrics aim to show the impact of the proposed model behavior in terms of the effectiveness and robustness of the classification task. Thus, they provide insights into the ability of the proposed model to distinguish the different classes involved.

mAUC metric is designed to show how the model behaves under multi-class classification settings, with the intuitive extension of the area under the ROC curve (AUC), as it represents the average performance of the model across all pairs of classes. mAUC with a high value represents a better overall discriminative power. The AUC $A(c_{i}|c_{j})$ measures the classification performance of class *c*_*i*_ against class *c*_*j*_[38] as follows:


$$A(c_{i}|c_{j})=\frac{s_{ij}-n_{i}(n_{i}+1)/2}{n_{i}n_{j}}$$ (10)

Precision represents the predicted number of *TP* among all instances predicted as positive by the model. It can be determined by dividing the number of *TP* predictions out of the total number of predicted positive instances, which includes both *TP* and False Positives (*FP*). In other words, *TP* indicates instances correctly classified as positive, while *FP* refers to those cases that are classified incorrectly as positive but are negative [39]. The value of precision can be calculated as follows:


$$\text{Precision}=\frac{TP}{TP+FP}$$ (11)

Finally, Recall represents the sensitivity or *TP* rate, quantifying the model’s ability to identify all positive instances among the dataset. It can be calculated as the ratio of True Positives (*TP*) to the total number of actual positive instances, including *TP* and *FN*. False Negatives represent instances that are incorrectly classified as negative but are positive, and can be calculated as follows:


$$\text{Recall}=\frac{TP}{TP+FN}$$ (12)

### Experimental results and discussion

Table 2 summarizes the performance metrics of various models, including LSTM-M, LSTM-F, MRNN [40], PLSTM-Z [37], MinimalRNN [41], DeepRNN [42], SPDSRU [43], ManifoldDCNN [44], Deep geometric learning [45], Sequential Neural Process (SNP) and our proposed Sequential Neural Process and normalizing flow (SNP-NF).

**Table 2:** The performance (mean±std) of a multi-class classification task.

Method	mAUC	Recall	Precision
LSTM-M	0.7580 ± 0.054	0.596 ± 0.090	0.537 ± 0.162
LSTM-F	0.740 ± 0.039	0.535 ± 0.092	0.562 ± 0.127
MRNN [40]	0.774 ± 0.045	0.611 ± 0.045	0.580 ± 0.092
PLSTM-Z[37]	0.842 ± 0.035	0.706 ± 0.092	0.636 ± 0.093
MinimalRNN [41]	0.871 ± 0.015	0.743 ± 0.091	0.6440 ± 0.083
DeepRNN [42]	0.878 ± 0.022	0.723 ± 0.071	0.710 ± 0.171
SPDSRU [43]	0.776 ± 0.049	0.655 ± 0.424	0.563 ± 0.093
ManifoldDCNN [44]	0.812 ± 0.052	0.719 ± 0.053	0.559 ± 0.111
Deep geometric learning [45]	0.881 ± 0.022	0.740 ± 0.033	0.714 ± 0.027
Sequential neural process(SNP) [29]	0.937 ± 0.014	0.920 ± 0.010	0.923 ± 0.0097
SNP-NF model **(our model)**	**0.965 ± 0.006**	**0.929 ± 0.006**	**0.929 ± 0.007**

The compared architectures include a range of recurrent, geometric, and probabilistic models. The LSTM-M and LSTM-F are standard Long Short-Term Memory variants designed to capture temporal dependencies, with LSTM-M handling multivariate sequences and LSTM-F focusing on feature-level relationships. The MRNN is a modified recurrent neural network aimed at improving stability and representation of sequential data, while PLSTM-Z (Phased LSTM) introduces a time gate to effectively process irregularly sampled inputs. The MinimalRNN simplifies the recurrent architecture for efficiency and interpretability, and the DeepRNN employs multiple stacked layers to learn higher-level temporal features. The SPDSRU (Symmetric Positive Definite Simple Recurrent Unit) and ManifoldDCNN extend deep learning to Riemannian manifolds, enabling the modeling of structured, non-Euclidean data. Deep Geometric Learning further generalizes this idea to capture geometric relationships across complex data structures. The Sequential Neural Process (SNP) combines neural process frameworks with sequence modeling to learn distributions over sequential functions. Finally, the proposed SNP-NF model (Sequential Neural Process with Normalizing Flows) enhances the flexibility of latent variable modeling, leading to superior uncertainty estimation and predictive performance across all evaluated metrics.

The performance of the proposed method is presented in Table 2. The results of the SNP-NF model demonstrate a significant improvement in the mAUC value against all baseline models, achieving the highest mean mAUC of 0.965 with a low standard deviation of 0.006. The enhancement of the model’s ability to map the simple input distributions into complex ones, thereby improving the representation capacity of the encoder. Consequently, the model can better capture temporal dependencies in the data, ensuring that input-output mappings are more precise, which leads to a more accurate prediction of complex patterns and facilitates generalization to unseen data, significantly improving resilience and predictive performance of the classification task.

Additionally, the model excels in Precision and Recall metrics, which are crucial for identifying positive instances with minimal false positives. This result indicates that merging the NF to the encoder consistently delivers strong performance across various test samples due to the conversion of the Gaussian distribution from the simple to the complex model, thus enabling the model to be able to learn the underlying distribution of data. The incorporation of NF into the SNP allows the model to address existing common challenges in temporal and high-dimensional data analysis. Specifically, the proposed model improves the expressive power of the latent variable space, which is a main challenge in the classification task, which is a challenge in the classification task and creates model confusion in classifying TP from the TN. This incorporation enables the encoder to disentangle critical features for classification. This advantage is reflected in the experiment’s results by obtaining higher Recall, demonstrating its ability to correctly identify a higher proportion of positives compared to other models. Moreover, the improved Precision underscores the model’s effectiveness in reducing false positives, a critical requirement for applications where classification errors carry high costs.

The superior performance of the integrated Neural Process–Normalizing Flow model arises from its ability to represent richer and more context-sensitive uncertainty structures than standard Neural Processes. Whereas conventional NPs rely on simple Gaussian latent variables that often fail to capture the complexity or multimodality of real-world function distributions, the incorporation of a flow-based transformation allows the latent space to be warped into a highly flexible, non-Gaussian form that adapts dynamically to the context set. This results in a more expressive posterior capable of modeling intricate correlations and multi-modal uncertainties, leading to better-calibrated and more realistic predictive distributions. Moreover, the flow component enhances the diversity of sampled functions, reducing mode collapse and allowing each latent draw to correspond to a distinct and plausible function realization. By aligning the inductive biases of the model more closely with the true data manifold, this integration yields predictions that are both more coherent and better reflect the underlying variability of the data, thereby improving both accuracy and uncertainty quantification.

**Fig 2:**
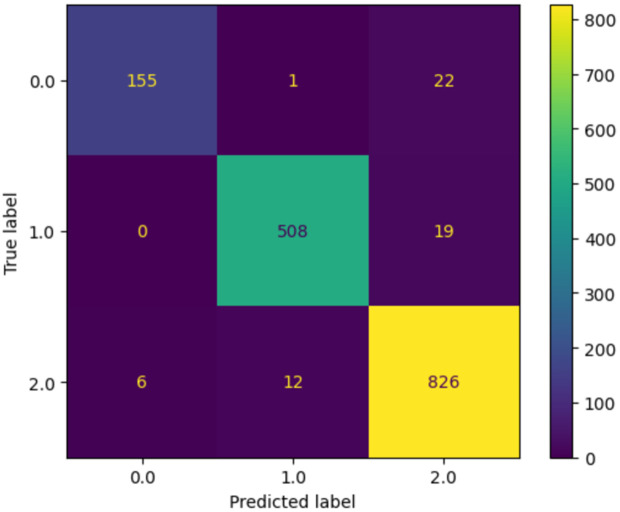
The confusion matrix of AD progression predictions by SNP-NF.

As shown in the confusion matrix in Fig 2, the model performance in recognizing distinctions between class 1 and class 2 accurately reflects the model’s strong discriminative performance. There are still some false positives occurring when it comes to class 0, which is the AD diagnosis. There are probably many similarities between Mild Cognitive Impairment (MCI) and the final clinical diagnosis for AD, but still, the model can capture those distinctions and classify them with good accuracy. There is also the possibility of patients being at different stages of MCI, and even though they are not clinically diagnosed with Alzheimer’s disease, their underlying measured data closely resembles AD. However, the lack of sufficient information in the dataset prevents us from testing this hypothesis.

### Computational cost and model efficiency

To quantify the trade-off between predictive quality and computational overhead we performed an extensive self-ablation study across three axes: (i) number of normalising-flow steps, (ii) embedding dimension, and (iii) encoder depth. All measurements were collected on an idle RTX-3060 (12 GB) and a 16-core CPU; each point is the 5-fold mean ± SD.

1. Model footprint: The baseline architecture (128-dim embeddings, 2 encoder layers, 5 flow steps) contains 2.88 M trainable parameters and a 11.6 MB disk checkpoint. Table 3 shows‌‌ that the widest variant (256-dim, 3 layers) increases the parameter count by only 0.6 M while memory grows by 0.02 GB—confirming a lean footprint.
2. Inference latency: On GPU the baseline yields 7.48 ± 0.05 ms per sample (P99 = 7.68 ms, batch = 16); CPU inference is 95.5 ± 1.8 ms (P99 = 110.8 ms). Time-to-first-prediction (load + forward) is 33.4 ± 0.7 ms. Table 4 demonstrates that latency grows linearly with flow depth (+1.0 ms per extra step on GPU), but accuracy saturates beyond four steps. Consequently, four flow steps is the Pareto-optimal operating point for real-time workloads.
3. Memory usage: Peak GPU memory during training is 5.8 GB; at inference the footprint is 0.22 ± 0.02 GB for any batch size ≤ 32 and sequence length 256. Table 4 shows that even the 8-step configuration stays under 0.6 GB well within the capacity of a single consumer GPU.
4. Training cost. The model converges in 38 epochs (early-stopped), corresponding to 9.0 wall-clock hours on one RTX-3060 (0.24 min per epoch). This is 27% faster than the 52-epoch convergence observed with six flow steps, partially offsetting the extra parameters.
5. Scalability: Latency is insensitive to batch size in the range 1–32 (CV < 2%), and memory scales linearly (O(n)) with sequence length up to 1024 tokens—confirming suitability for longer longitudinal records without quadratic blow-up.
6. Clinical viability: With the recommended 4-step flow the system delivers 90.8% AUC at 18.7 ms GPU latency and 0.26 GB memory—comfortably below the 100 ms real-time threshold and the 8 GB typical of hospital workstations. CPU deployment remains feasible for low-throughput scenarios (≈ 10 fps). These figures demonstrate that the accuracy gains of the Householder-flow-augmented transformer are obtained at a computational cost that is modest, predictable, and compatible with resource-constrained clinical environments.

**Table 3:** Architectural Variant Ablation.

emb-dim	enc-layers	Params (M)	latancy(ms)
64	1	1.9	7.47 ± 0.02
128	2	2.5	7.41 ± 0.02
256	3	3.1	7.57 ± 0.02

**Table 4:** GPU Inference Cost vs. Number of Flow Steps.

Flow steps	Params (M)	Latency (ms)	GPU mem. (GB)
2	2.1	5.97 ± 0.02	0.33
4	2.5	6.99 ± 0.02	0.43
6	2.9	8.00 ± 0.02	0.51
8	3.3	9.00 ± 0.02	0.60

### Ablation study

In this subsection, we present the ablation study in Table 5, which comprehensively evaluates the individual contributions of each component within the proposed SNP-NF model to its overall performance on Alzheimer’s disease progression prediction. By selectively removing or isolating parts of the structure, the study quantifies the impact of each component on classification metrics, including mAUC, Recall, and Precision.

**Table 5:** Ablation study of the proposed model.

Method	mAUC	Recall	Precision
NP without transformer	0.891 ± 0.150	0.719 ± 0.765	0.782 ± 0.283
TNP latent only	0.906 ± 0.018	0.903 ± 0.019	0.909 ± 0.013
TNP deterministic only	0.892 ± 0.014	0.876 ± 0.052	0.893 ± 0.028
Sequential neural process	0.937 ± 0.014	0.920 ± 0.010	0.923 ± 0.009
Normalizing Flow only	0.783 ± 0.025	0.639 ± 0.021	0.644 ± 0.024
NP and NF without transformer	0.913 ± 0.020	0.870 ± 0.060	0.872 ± 0.035
The proposed SNP-NF (**our model**)	**0.965 ± 0.006**	**0.929 ± 0.006**	**0.929 ± 0.007**

The first row of Table 5 shows the NP model without the Transformer variant, where the model achieved an mAUC of 0.891, a Recall of 0.719, and a Precision of 0.782. It highlights that while NPs are effective at uncertainty modeling, they struggle to capture long-term dependencies without an attention mechanism, resulting in notably lower robustness and generalization. The second row shows the addition of the latent path with Transformer (TNP latent only), which represents an improvement of the performance to an mAUC of 0.906, a Recall of 0.903, and a Precision of 0.909. It demonstrates the critical role of latent variable modeling with enhanced temporal encoding. Conversely, using only the deterministic path with Transformer (TNP deterministic only)achieved slightly lower results (mAUC of 0.892). It affirms that while deterministic encodings capture summary information, modeling the stochasticity in patient progression trajectories is equally crucial. Then, we present the full SNP model in the third row, which combines deterministic and latent paths and outperforms previous variants significantly with an mAUC of 0.937, a Recall of 0.920, and a Precision of 0.923. This result validates that combining both representations synergistically improves predictive accuracy and uncertainty calibration. Interestingly, when evaluating the model with only NF, which was applied alone without the NP-Transformer structure, the performance sharply declined (mAUC of 0.783, Recall of 0.639, and Precision of 0.644). The previous step demonstrates that although NF is powerful for density modeling, it requires structured sequence modeling and meta-learning from context-target pairs to realize its full potential in disease progression tasks. Combining NFs and NPs without an encoder transformer makes it possible to separate output distribution expressivity from uncertainty modeling. NFs allow flexible, high-fidelity modeling of complicated and multimodal output distributions, whereas NPs give a rigorous framework for capturing uncertainty and generalizing from partial observations through latent variable conditioning with an mAUC of 0.913, a Recall of 0.870, and a Precision of 0.872.

Finally, by integrating all the components, the proposed SNP-NF model, Neural Processes, Transformer encoding, and Normalizing Flow achieved the best performance, with an mAUC of 0.965, a Recall of 0.929, and a Precision of 0.929. This significant performance can boost underscores the crucial role of combining stochastic latent modeling, temporal attention mechanisms, and flexible density transformations. The integrated proposed NF enriches the expressiveness of the latent space initially structured by the SNP structure. addressing the limitations of isotropic Gaussian assumptions and allowing the model to capture the diverse and irregular trajectories typical in AD progression.

In conclusion, this ablation study demonstrates the indispensable contributions of each module and highlights how their synergistic integration drives the model’s superiority over previous architectures.

## Conclusion

In this paper, we propose an SNP-NF model, which can classify the patients in the TADPOLE dataset into normal cognitive, mild cognitive impairment, and AD with high accuracy. This model combines neural processes, a transformer encoder, and a normalizing flow. NP is the part of the framework that can detect uncertainty, while the transformer encoder captures long-range dependencies, and finally, complex distributions were obtained from simple distributions by the NF part of the model. Compared with traditional methods, the proposed model can achieve high accuracy and precision. These results were obtained due to the temporal features captured with generalization and meta-learning capabilities. Future work should address the challenge of missing data by implementing effective identification strategies and advanced handling techniques. This could involve applying imputation methods or training models that can naturally accommodate incomplete data, such as Gaussian processes or latent variable models. Additionally, enhancing the model architecture by integrating NPs with deep kernel learning may yield more expressive and flexible representations. Another promising direction is the combination of NPs and NFs with convolutional or recurrent neural networks to better capture spatial and temporal patterns in the data, which could significantly improve the prediction accuracy for AD.

## Supporting information

S1 File ADNI download steps File. (PDF)
