# Supplementary material for: Normalizing flow based neural processes for Alzheimer’s disease progression prediction
Source: PLoS One. 2026 Apr 20;21(4):e0345958. doi: 10.1371/journal.pone.0345958 (PMC13095108; doi:10.1371/journal.pone.0345958)

Application Review

The ADNI Data Sharing and Publications Committee (DPC) generally reviews data use applications within two weeks of submission. Each application is carefully reviewed to verify investigator's affiliation with a scientific or educational institution and on the basis of the proposed research or data use. Incomplete applications or those without a clear focus will not receive approval. The results of the Committee's review will be sent via email. Approved applicants will receive login information to access and download ADNI data from the LONI Image and Data Archive (IDA).

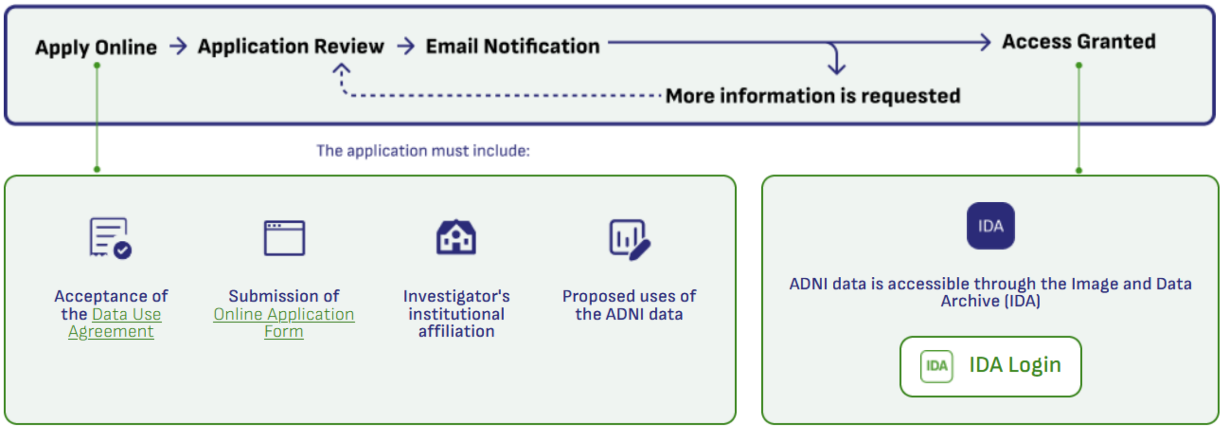

The steps after Login IDA:

1- From the select Tab we choose the ADNI button.

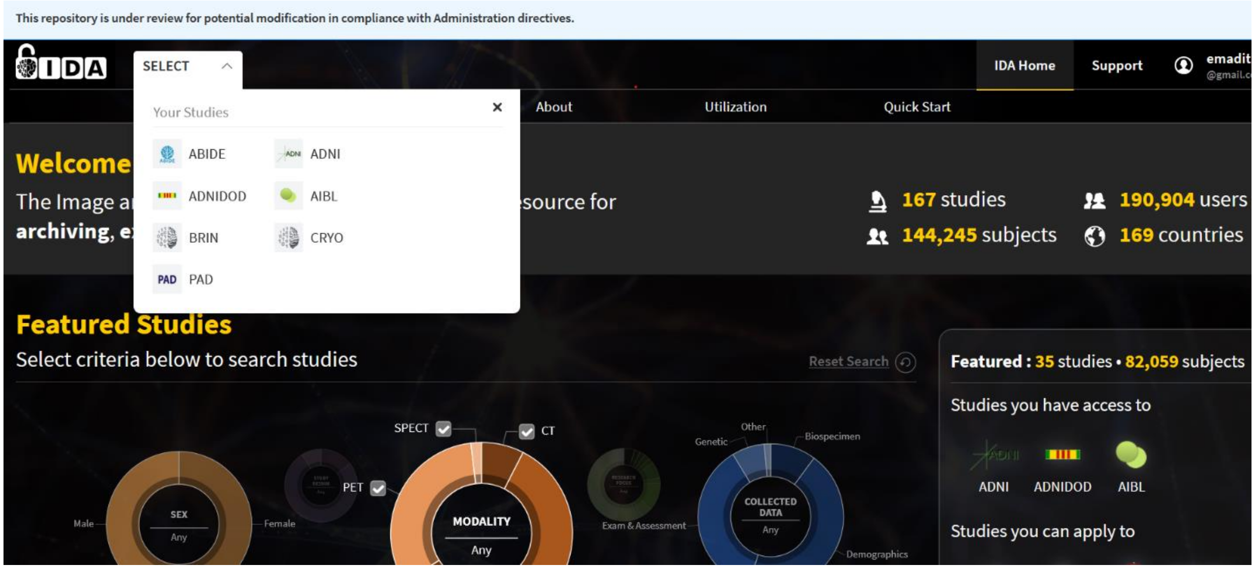

2-From search and download tab choose the study file tab.

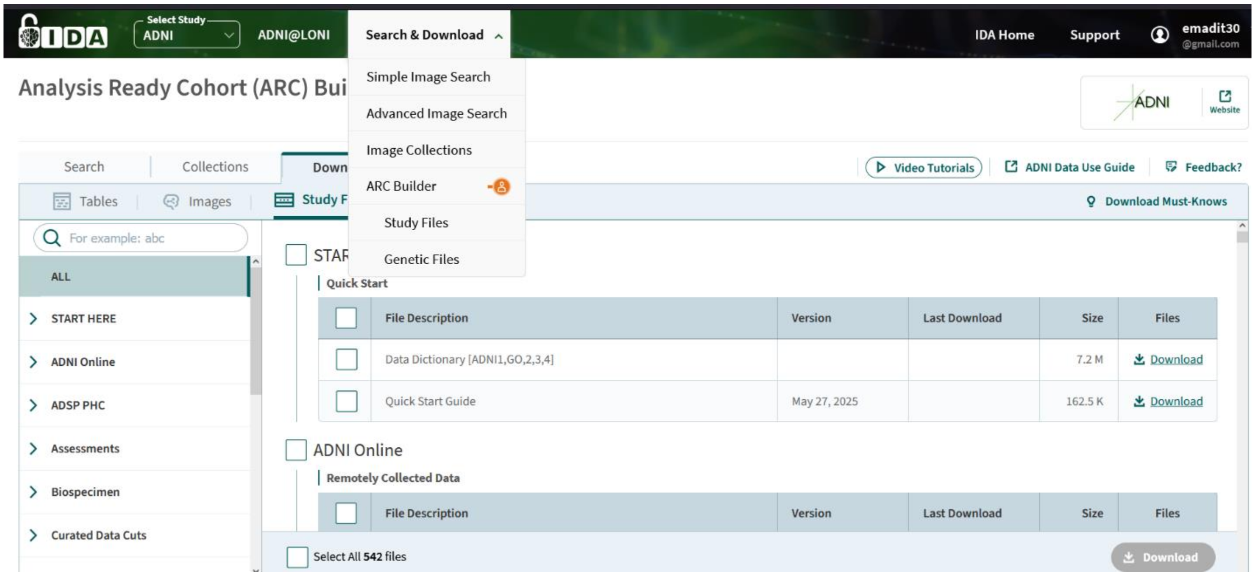

3-From the left side choose the TEST DATA then choose the Data for Challenges then from the right side choose the Tadpole Challenge Data then choose download.

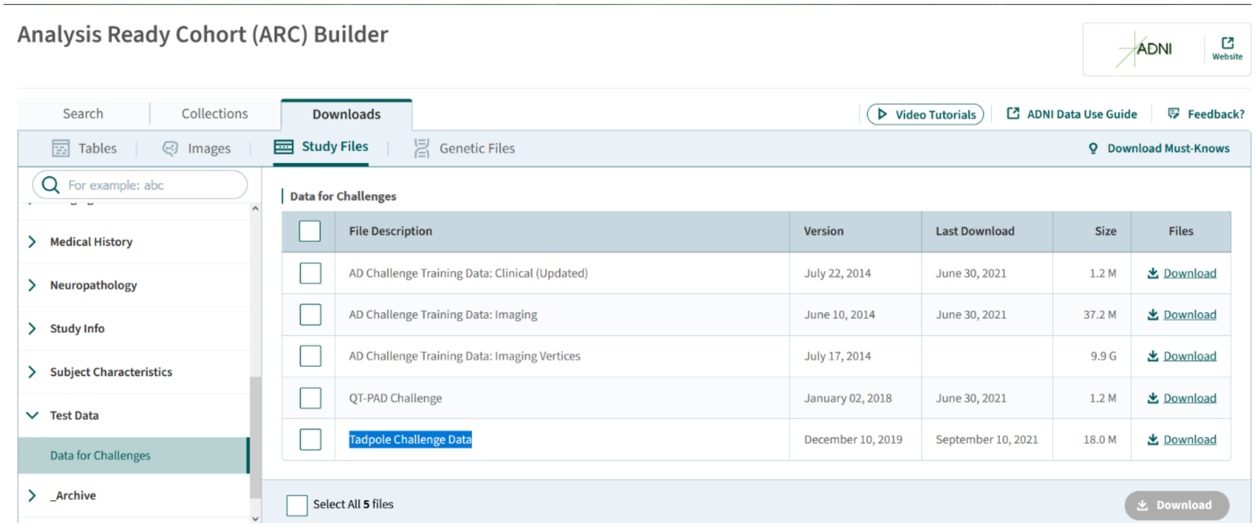

Supplement: S1 File — (PDF) [file pone.0345958.s001.pdf]
